# BEX2 suppresses mitochondrial activity and is required for dormant cancer stem cell maintenance in intrahepatic cholangiocarcinoma

**DOI:** 10.1038/s41598-020-78539-0

**Published:** 2020-12-09

**Authors:** Keiichi Tamai, Mao Nakamura-Shima, Rie Shibuya-Takahashi, Shin-Ichiro Kanno, Akira Yasui, Mai Mochizuki, Wataru Iwai, Yuta Wakui, Makoto Abue, Kuniharu Yamamoto, Koh Miura, Masamichi Mizuma, Michiaki Unno, Sadafumi Kawamura, Ikuro Sato, Jun Yasuda, Kazunori Yamaguchi, Kazuo Sugamura, Kennichi Satoh

**Affiliations:** 1 Division of Cancer Stem Cell, Miyagi Cancer Center Research Institute, 47-1 Nodayama, Medeshima-Shiode, Natori, Miyagi 981-1293 Japan; 2 Division of Molecular and Cellular Oncology, Miyagi Cancer Center Research Institute, 47-1, Medeshima-Shiode, Natori, Miyagi Japan; 3 IDAC Fellow Research Group for DNA Repair and Dynamic Proteome Institute of Development, Aging and Cancer (IDAC), Tohoku University, Sendai, 980-8575 Japan; 4 Department of Gastroenterology, Miyagi Cancer Center, 47-1, Medeshima-Shiode, Natori, Miyagi Japan; 5 Department of Surgery, Miyagi Cancer Center, 47-1, Medeshima-Shiode, Natori, Miyagi Japan; 6 Department of Surgery, Tohoku University Graduate School of Medicine, 1-1, Seiryo-cho, Aoba-ku, Sendai, Miyagi Japan; 7 Department of Urology, Miyagi Cancer Center, 47-1, Medeshima-Shiode, Natori, Miyagi Japan; 8 Department of Pathology, Miyagi Cancer Center, 47-1, Medeshima-Shiode, Natori, Miyagi Japan; 9 Division of Gastroenterology, Tohoku Medical and Pharmaceutical University, 1-15-1, Fukumuro, Miyagino-ku, Sendai, Miyagi Japan; 10 Division of Hepatobiliary and Pancreatic Surgery, Tohoku Medical and Pharmaceutical University, 1-15-1, Fukumuro, Miyagino-ku, Sendai, Miyagi Japan

**Keywords:** Cancer, Cancer stem cells, Gastrointestinal cancer

## Abstract

Cancer stem cells (CSCs) define a subpopulation of cancer cells that are resistant to therapy. However, little is known of how CSC characteristics are regulated. We previously showed that dormant cancer stem cells are enriched with a CD274^low^ fraction of cholangiocarcinoma cells. Here we found that BEX2 was highly expressed in CD274^low^ cells, and that BEX2 knockdown decreased the tumorigenicity and G_0_ phase of cholangiocarcinoma cells. BEX2 was found to be expressed predominantly in G_0_ phase and starvation induced the USF2 transcriptional factor, which induced BEX2 transcription. Comprehensive screening of BEX2 binding proteins identified E3 ubiquitin ligase complex proteins, FEM1B and CUL2, and a mitochondrial protein TUFM, and further demonstrated that knockdown of BEX2 or TUFM increased mitochondria-related oxygen consumption and decreased tumorigenicity in cholangiocarcinoma cells. These results suggest that BEX2 is essential for maintaining dormant cancer stem cells through the suppression of mitochondrial activity in cholangiocarcinoma.

## Introduction

Cholangiocarcinoma is the most common primary malignancy of the biliary tract and one of the most difficult intra-abdominal malignancies to treat. Surgical management is the only potentially curative treatment, but is limited to early-stage disease. Chemotherapy (cisplatin or gemcitabine) and radiation therapy are of limited effectiveness in cholangiocarcinoma, because of its desmoplastic stroma and genetic heterogeneity^1^. Recently, targeted therapy has been applied to clinical treatment for several types of cancer. For example, an anti-EGFR (epidermal growth factor receptor) antibody had a dramatic response on lung cancer with EGFR mutations^2^. However, no effective target molecule has been discovered for cholangiocarcinoma.

“Cancer stem cell theory” refers to a mechanism of heterogeneous cancer tissue formation^3^. Cancer stem cells (CSCs) possess several unique properties, such as quiescence, therapy resistance, and tumorigenicity, which are related to a poor prognosis. Cancer stem cells share several key properties with normal stem cells^4^ but only CSCs are able to initiate tumors because they are solely capable of self-renewal and unlimited replication. CSCs are also resistant to chemotherapy because of their infrequent replication^5^. We previously reported that CD274, also known as PD-L1, suppresses CSC phenotypes in cholangiocarcinoma. CD274-knockdown in cholangiocarcinoma cell lines resulted in increased tumorigenicity and aldehyde dehydrogenase (ALDH) activity, and CD274^low^ cells are present in G_0_ phase^6^, suggesting that a CD274^low^ fractions are enriched with dormant cancer stem cells. However, the precise molecular mechanisms of how the CSC-phenotype is maintained are poorly understood in cholangiocarcinoma.

The BEX2 (brain expressed X-linked gene 2) gene is mapped on the mouse X chromosome and human Xq22^7^, and encodes an approximately 20-kDa protein detected in some types of cancer. In breast cancer, BEX2 plays a critical role in c-Jun/JNK pathways for the cell cycle^8,9^. In U251 astrocytoma and U87 glioma cell lines, BEX2 is essential for migration and invasion^10,11^. In colon cancer, BEX2 promotes tumor proliferation^12^, while Foltz et al.^13^ reported that BEX2 is silenced in brain tumors. Thus, the precise function of BEX2 is controversial and little is known about what molecule(s) interact with BEX2 in cancer. A previous report suggested that BEX2 is expressed in stem/progenitor cells of the liver, pyloric stomach, and hematopoietic cells in mice^14^. Recently, BEX2 was shown to be important for reprogramming induced pluripotent stem (iPS) cells^15^. These data suggest a significant role for BEX2 in stem cells.

Here, we screened differentially expressing genes in a CSC-enriched fraction in cholangiocarcinoma and identified BEX2 as a key molecule for maintaining dormant CSCs.

## Results

### BEX2-mediated cancer stem cell properties

**Figure 1:**
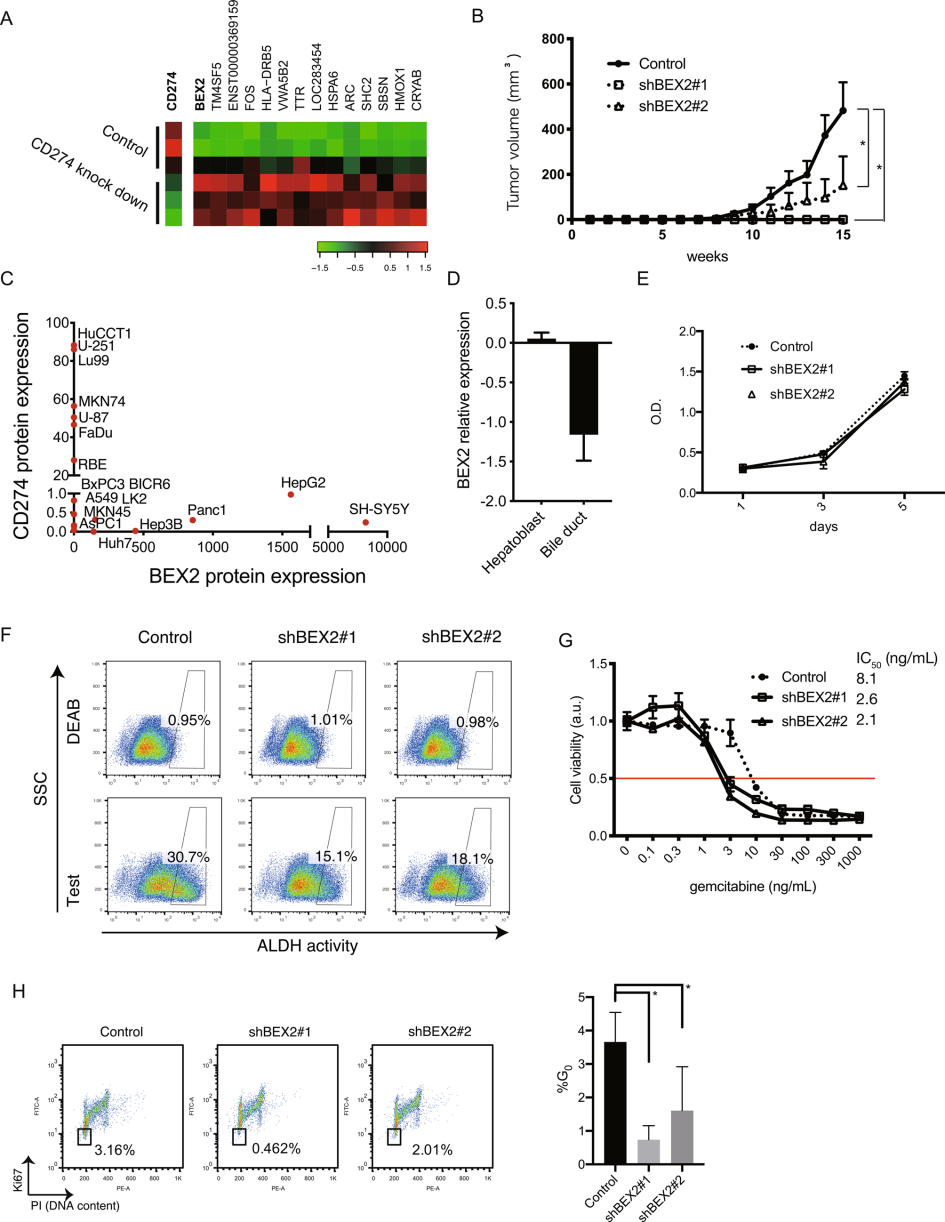
Identification of BEX2 as a cancer stem cell-related molecule. (**A**) Heatmap of gene expression microarray analysis. CD274-knockdown and control RBE cells were used and gene expression data were analyzed by a rank product method. (**B**) Tumorigenicity assay of HuCCT1 control and BEX2-knockdown cells (10^2^ cells per site) were subcutaneously injected into NOD/SCID/γc^null^ (NOG) mice and tumor development over time. Control: n = 12, shRNA#1 and shRNA#2: n = 6. *P < 0.05. (**C**) Expression levels of CD274 and BEX2 in several cancer cell lines. CD274 was determined by mean intensity of flow cytometry analysis and BEX2 was detected by western blotting analyzed using ImageJ software. (**D**) Expression of BEX2 mRNA in the mouse hepatoblast cell line (HBC-3) and differentiated cells. Data were obtained from Gene Expression Omnibus (GDS970). (**E**) MTT assays using BEX2-knockdown cells. n = 5. (**F**) ALDEFLUOR assay using HuCCCT1 control and BEX2-knockdown cells. ALDH-positive gates were determined with N,N-diethylaminobenzaldehyde (DEAB, an ALDH inhibitor) treatment. SSC, side scatter. Test, assay without DEAB treatment. (**G**) Gemcitabine sensitivity in HuCCT1 control and BEX2-knockdown cells determined by MTT assay. n = 3. IC, inhibitory concentration. (**H**) (left panel) Representative graphs of cell cycle assay using HuCCT1 cells fixed with 70% ethanol and stained with PI and Ki67 followed by flow cytometry. (right panel) Summary of the percentage of G_0_ population (n = 3, *P < 0.05).

To investigate gene(s) responsible for dormant cancer stem cells enriched in the CD274^low^ fraction of cholangiocarcinoma cells, we screened genes differentially expressed between CD274-knockdown and control RBE cholangiocarcinoma cells. Several genes including BEX2 were significantly up-regulated in the CD274-knockdown cells (Fig. 1A) and we therefore, focused on BEX2 because of the attenuation of tumorigenicity by knock down using shRNA (Fig. 1B). To confirm that BEX2 and CD274 are mutually exclusive in their expression, we tested BEX2 and CD274 protein expression by western blot or flow cytometry using 16 cell lines derived from various cancers, including cholangiocarcinoma, hepatocellular carcinoma, gastric cancer, lung cancer, and glioma. Similar to the RBE cell line, in the cholangiocarcinoma cell line HuCCT1, BEX2 or CD274 expression was mutually exclusive (Fig. 1C). In a gene expression database (Gene Expression Omnibus, GDS970), BEX2 is expressed in murine hepatoblasts and downregulated in mature bile ducts (Fig. 1D), suggesting that BEX2 is involved in liver stem cell function.

**Figure 2:**
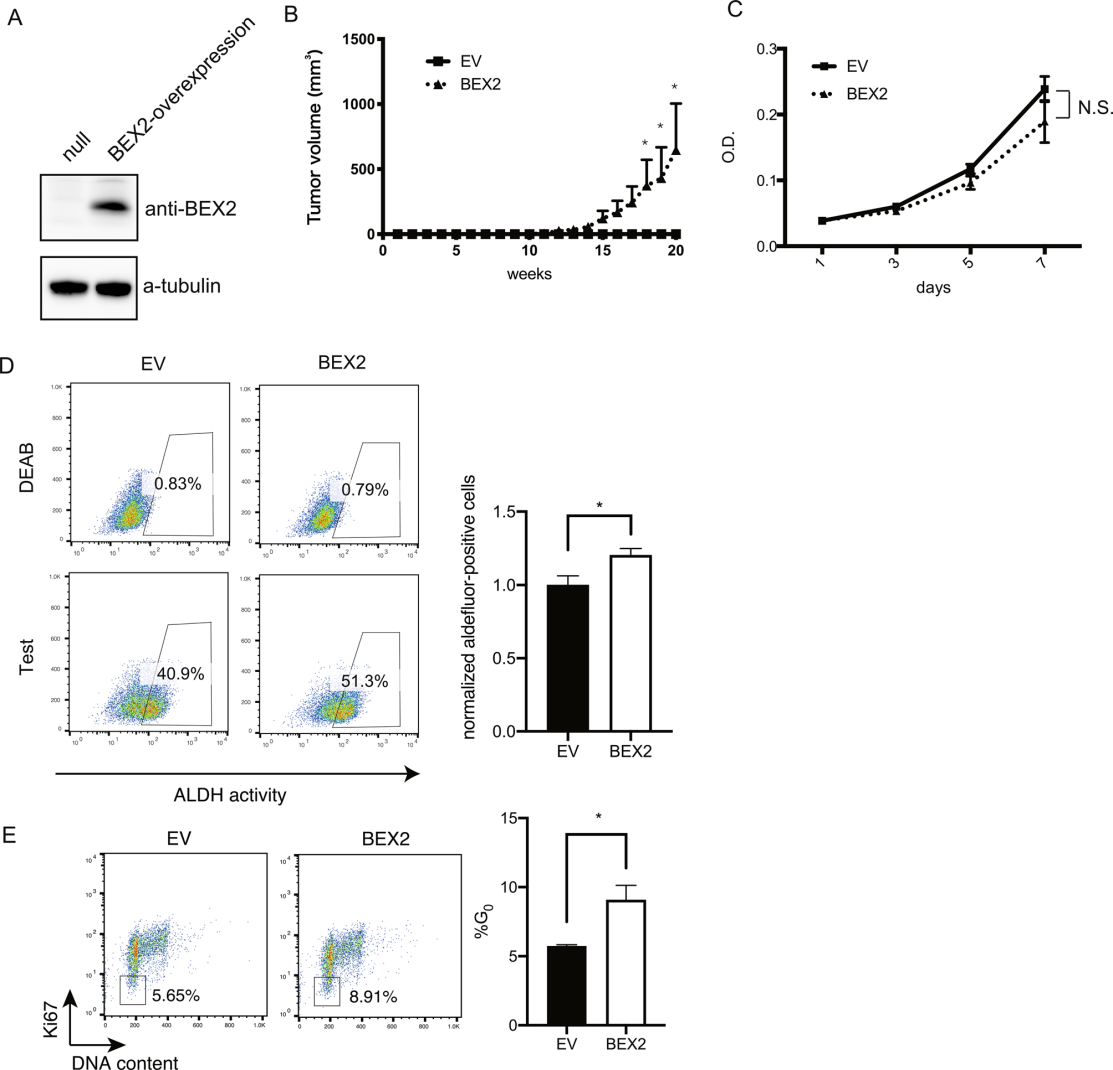
Overexpression of BEX2 increased dormant cancer stem cells. (**A**) Western blotting analysis of control and BEX2-overexpressing HuCCT1 cells. (**B**) Tumorigenicity assay of BEX2-overexpressing and control HuCCT1 cells. A total of 10^2^ HuCCT1-BEX2 or control cells were subcutaneously injected into NOG mice and the diameter of tumors was measured; n = 6. *P < 0.05. (**C**) Cell proliferation curve of BEX2-overexpressing and control HuCCT1 cells was determined by MTT assay; n = 3. N.S., not significant. (**D**) ALDH activity was determined with an ALDEFLUOR assay in BEX2-overexpressing and control HuCCT1 cells. ALDH activity was increased in BEX2-overexpressing HuCCT1 cells. ALDH-positive gates were determined with DEAB treatment. SSC, side scatter. Test, assay without DEAB treatment. (left panel) Representative graphs of ALDH assay; (right panel) summary of the positivity of ALDH. *P < 0.05. (**E**) Cell cycle assay of HuCCT1 cells cultured in medium containing 0.1% FBS for 24 h, and then fixed with 70% ethanol and stained with PI and Ki67 for flow cytometry sorting. G_0_ phase (open square) was increased in BEX2-overexpressing HuCCT1 cells. (left panel) Representative graphs of cell cycle analysis; (right panel) summary of the percentage of G_0_ fraction. *P < 0.05.

**Figure 3:**
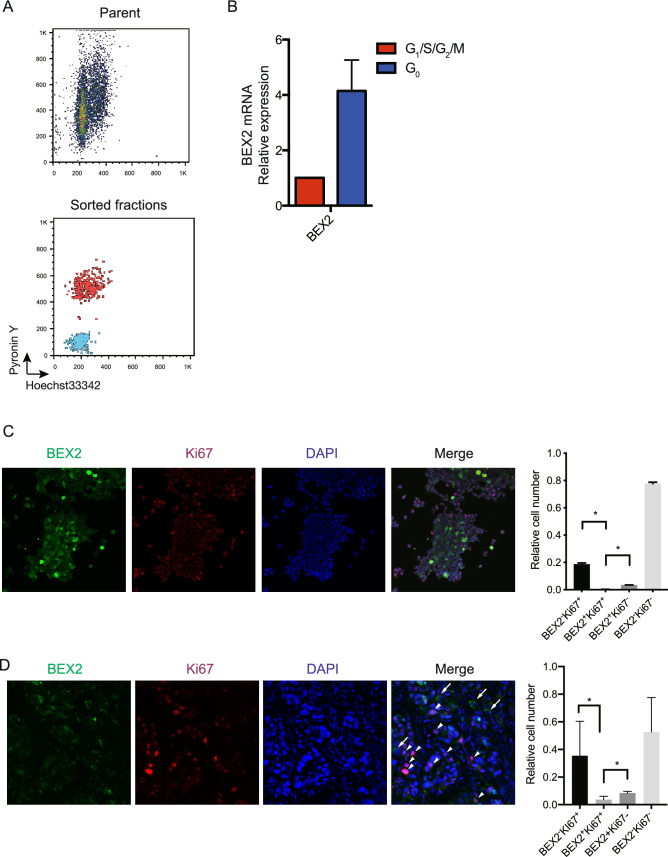
BEX2 is predominantly expressed in dormant cancer cells. (**A**) Representative data of flow cytometry analysis of HuCCT1 cells. HuCCT1 cells (upper panel) stained with pyroninY and Hoechst33342. Sorted fractions (blue: G_0_, red: G_1_/S/G_2_/M) were re-analyzed by flow cytometry. (**B**) Real-time PCR of sorted HuCCT1 cells. (**C**) Immunocytochemistry of 293 T-FlpIn-T-REx-Flag-BEX2 cells. Cells were stained with anti-Flag (green), anti-Ki67 (magenta), and DAPI (blue). Quantification of stained cell number is shown in the right panel (*P < 0.05). (**D**) (Left panel) Immunohistochemistry of CHOL4 cells (a PDX-derived cell line) xenografted into NOG mice. Green: BEX2, Magenta: Ki67, Blue: DAPI. Arrows indicate BEX2^+^Ki67^–^ cells and arrowheads indicate BEX2^–^Ki67^+^ cells. (Right panel) Quantification of stained cell number is shown (a total of 434 cells were counted in four randomly selected areas, *P < 0.05).

To examine whether BEX2 is involved in cancer stem cell properties, we established two HuCCT1 BEX2-knockdown cell sublines, which expressed minimal BEX2 as indicated by real-time PCR and western blotting analysis (Supp Fig. 1A and B). The BEX2-knockdown HuCCT1 cells and control cells (cells expressing short hairpin RNA against luciferase) were examined for their tumorigenic activities in NOG mice. BEX2-knockdown line significantly suppressed HuCCT1 tumorigenicity (Fig. 1B and Supplemental Fig. 2A), although no difference was found between the BEX2-knockdown and control cells in in vitro cell proliferation (Fig. 1E). These results suggest that BEX2 is required for the tumorigenic activity of HuCCT1 cells. We then measured aldehyde dehydrogenase (ALDH) activity, which is known as a cancer stem cell marker, by using the ALDEFLUOR assay. BEX2-knockdown lines had lower ALDH activity than the control (Fig. 1F). We got the same results in RBE cell line (Supp. Fig. 3B). We also tested if the BEX2 knockdown lines had an altered gemcitabine sensitivity and found that the BEX2-knockdown cells were more sensitive to gemcitabine than the control cells (Fig. 1G). Given that one of the hallmarks of cancer stem cells is their dormant phase, we examined the cell cycle in BEX2-knockdown cell lines. We found that G_0_, defined by Ki67^low^ and weak propidium iodide (PI) labeling, was decreased in the BEX2-knockdown cells compared to that in control cells (Fig. 1H). The same results were obtained using RBE cell line (Supp. Fig. 3C). Microarray analysis also showed that the BEX2-knockdown cells had altered cell cycle-related pathways (Table 1). Collectively, we conclude that BEX2 is potentially required for the maintenance of dormant cancer stem cells. To confirm this hypothesis, we investigated the phenotypes of dormant CSCs using BEX2-overexpressing cells (Fig. 2A). In an in vivo tumorigenicity assay, BEX2-overexpressing cells (HuCCT1-BEX2) had higher tumorigenic activity compared to empty vector-expressing cells (Fig. 2B), although in vitro proliferation was slightly decreased in BEX2-overexpresseion cells (Fig. 2C). ALDH activity also increased in the BEX2-overexpresseion cell line (Fig. 2D). Furthermore, the G_0_ phase increased in BEX2-overexpressing cells in starvation conditions (Fig. 2E). Based on these results, we hypothesized that BEX2 is critically involved in the G_0_ phase; therefore, we measured BEX2 expression in G_0_. When cells were sorted into G_0_ and other cell cycle phases, BEX2 was expressed predominantly in the G_0_ phase in HuCCT1 and RBE cell lines (Fig. 3A,B, and Supp. Fig. 3D). We also investigated the pattern of BEX2 protein expression using immunocytochemistry and found that BEX2 and the proliferation marker, Ki67, were expressed in 293 T-FlpIn-T-REx-Flag-BEX2 cells in a mutually exclusive manner (Fig. 3C). As HuCCT1 cells could proliferate but cannot develop heterogenous tissue in mice, we stained newly established cholangiocarcinoma-xenografted samples (CHOL4) with anti-BEX2 and anti-Ki67 antibody and got similar results (Fig. 3D). These data suggest that BEX2 plays important roles in the G_0_ phase of the cell cycle.

**Table 1:** Gene ontology assessment of differentially expressed genes from microarray data comparing control and BEX2-knockdown HuCCT1 cells analyzed by ToppGene (https://toppgene.cchmc.org/).

Ranking			*P*-value
1	GO:0022403	Cell cycle phase	7.04E−18
2	GO:0022402	Cell cycle process	1.06E−17
3	GO:0000278	Mitotic cell cycle	1.15E−17
4	GO:0006260	DNA replication	4.12E−17
5	GO:0007049	Cell cycle	5.13E−17
6	GO:0006259	DNA metabolic process	2.14E−15
7	GO:0051325	Interphase	2.78E−11
8	GO:0051329	Interphase of mitotic cell cycle	4.38E−11
9	GO:0022616	DNA strand elongation	4.23E−10
10	GO:0044427	Chromosomal part	4.42E−10

### Transcriptional regulation and proteasomal degradation of BEX2

**Figure 4:**
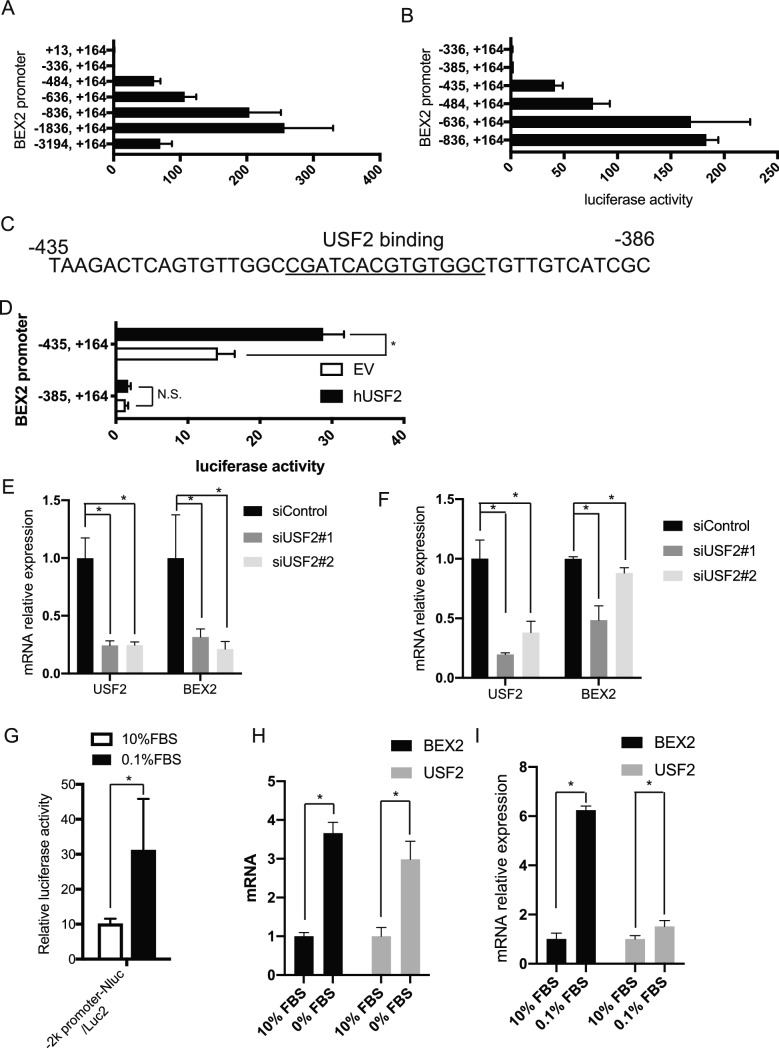
Identification of USF2 as a transcription factor of BEX2. (**A**,**B**) Luciferase assay using 293 T cells transfected with pNL1.1-Nluc-Neo expressing the indicated BEX2 promoter regions. (**C**) A region of the BEX2 promoter sequence. Underlined sequence is the predicted binding site of USF2. (**D**) Luciferase assay of 293 T cells transfected with the indicated promoter region in pNL1.1-Nluc-Neo vector, and pcDNA4-hUSF2-myc or empty vector (EV). (**E**,**F**) Real-time PCR of HuCCT1 (**E**) and RBE (**F**) cells. The cells were knocked down using siRNA against human USF2. After 3 days, cells were harvested and total RNA was extracted. (**G**) Luciferase activity assay of pNL1.1-BEX2promoter2k-Nluc-Neo was transfected into 293 T cells that were starved for 24 h. pGL4.10[luc2] was co-transfected into 293 T cells as a control. (**H**,**I**) Real-time PCR. HuCCT1 (**H**) or RBE (**I**) cells that were starved for 96 h and 48 h, respectively, before mRNA expression was measured. All the bars indicate mean ± SD. ^*^P < 0.05. N.S., not significant.

Next, to clarify how BEX2 is regulated and expressed in the G_0_ phase, we tried to identify the promoter region of the BEX2 gene. Approximately 3 kb upstream of the BEX2 coding region was cloned and used in a luciferase assay. We found that the region from − 385 to − 435 was critical for the transcription of luciferase (Fig. 4A,B). We searched for transcriptional factor the binding sites in this region using the ENCODE database (https://genome.ucsc.edu/ENCODE/index.html) and the transcription factor USF2 can bind to the CGATCACGTTGTGGC motif in this region (Fig. 4C). To confirm the function of USF2 at the BEX2 promoter, we performed the luciferase assay with 293 T cells overexpressing USF2. Overexpression of USF2 caused a clear increase of luciferase activity (Fig. 4D). We also tested whether USF2 controls BEX2 transcription. The knock down of USF2 resulted in a clear reduction of BEX2 mRNA in HuCCT1 (Fig. 4E) and RBE (Fig. 4F) cells. Serum-starved conditions generally induce a reduction in cell proliferation and quiescence^16^. We observed an upregulation in promoter activity in cells expressing the 2-kb sequence upstream of the BEX2 coding region in serum-starved conditions (0.1% FBS) (Fig. 4G and Supplemental Fig. 2B). Moreover, both BEX2 and USF2 were upregulated under this growth condition (Fig. 4H,I, and Supplemental Fig. 2B), indicating that USF2 promotes BEX2 expression at the transcriptional level in serum-starved condition.

**Figure 5:**
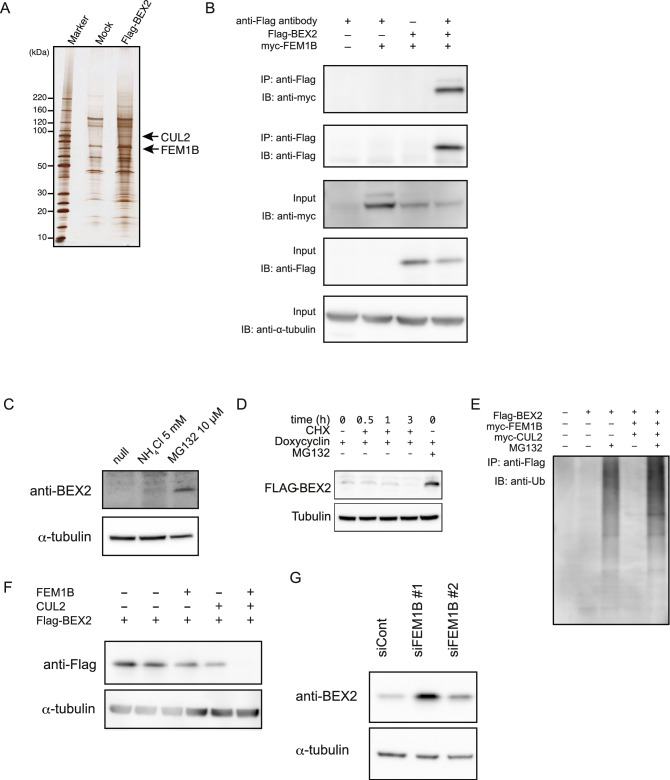
BEX2 is degraded by a proteasomal-dependent pathway. (**A**) BEX2-binding partners were determined by LC/MS analysis. Immunoprecipitation (IP) was performed using 293 T-FlpIn-T-REx-Flag-BEX2 cells. (**B**) Immunoprecipitation of FEM1B using anti-Flag antibody. Flag-BEX2 and myc-FEM1B were transfected into 293 T cells, and IP was performed. (**C**) HuCCT1 cells were stimulated with the lysosomal inhibitor (NH_4_Cl) or proteasomal inhibitor (MG132) and the expression level of BEX2 was determined by western blot analysis. (**D**) The degradation rate of Flag-BEX2 in 293 T-FlpIn-T-REx-Flag-BEX2 cells was determined by western blot analysis. CHX, cycloheximide. (**E**) Immunoprecipitation assay of 293 T cells transfected with the indicated plasmids and immunoprecipitated with an anti-Flag antibody showed that Flag-BEX2 was ubiquitinated under the co-transfection of FEM1B and CUL2. (**F**) 293 T cells were transfected with the indicated plasmids and the expression level of Flag-BEX2 was determined by western blot analysis. (**G**) FEM1B expression was knocked-down by targeted siRNA in HuCCT1-BEX2 cells. Two days after transfection, cells were harvested and BEX2 expression was determined by western blotting.

We conducted a comprehensive screen of BEX2-binding proteins by immunoprecipitation using 293 T-FlpIn-T-REx-Flag-BEX2 cells. Proteins specifically bound to BEX2 were identified by nanoLC/MS/MS. FEM1B and CUL2, which are a substrate recognition subunit and a ubiquitin ligase in an E3 complex, respectively^17^, were bound to BEX2 (Fig. 5A). Thus, we also performed immunoprecipitation using BEX2- and FEM1B-overexpressing 293 T cells to confirm the binding between BEX2 and FEM1B (Fig. 5B). Indeed, the amount of BEX2 was increased by treatment with MG132, a well-known proteasomal inhibitor, but not by treatment with the lysosomal inhibitor, NH_4_Cl (Fig. 5C). These results suggest that BEX2 is degraded in a proteasome-dependent manner. In 293 T cells, BEX2 was degraded completely within 3 h after treatment with cycloheximide, a well-known protein synthesis inhibitor (Fig. 5D). BEX2 was also ubiquitinated and the degree of ubiquitination increased with overexpression of FEM1B and CUL2 (Fig. 5E). To clarify the role of the E3 complex, FEM1B and CUL2 were introduced into 293 T cells and BEX2 protein expression was measured. BEX2 was almost completely degraded when both FEM1B and CUL2 were overexpressed (Fig. 5F). Furthermore, we examined whether BEX2 expression was altered by the knockdown of FEM1B. FEM1B knockdown increased BEX2 expression in HuCCT1-BEX2 cells (Fig. 5G). These data indicate that BEX2 proteins are degraded by a E3 complex that contains FEM1B and CUL2.

### BEX2-mediated suppression of mitochondrial activity

**Figure 6:**
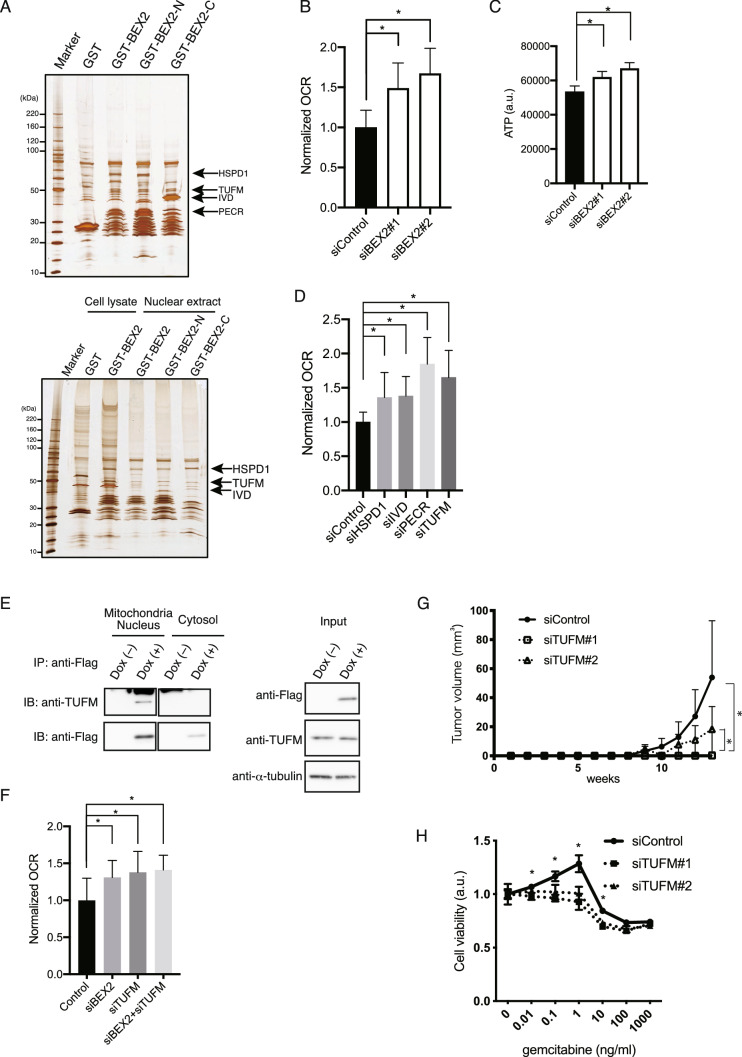
BEX2 binds TUFM and suppresses in mitochondrial activity. (**A**) BEX2-binding partners were evaluated by LC/MS in both mouse kidney (upper panel) and HuCCT1 cell (lower panel) lysates, respectively. These results indicate that mitochondria-related proteins HSPD1, TUFM, IVD, and PECR (arrows) are direct binding partners of BEX2. (**B**) Oxygen consumption ratio (OCR, pmol/min) was determined in control and BEX2-knockdown HuCCT1 cells using a Flux analyzer. The data was normalized to relative cell numbers. *P < 0.05. (**C**) ATP level was determined by Cell Titer Glo in control and BEX2-knockdown HuCCT1 cells under glucose-free RPMI medium for 1 h. a.u., arbitrary units. n = 5, *P < 0.05. (**D**) Oxygen consumption ratio (OCR) was determined in control and knockdown lines of BEX2-binding partners in HuCCT1 cells using a Flux analyzer. The data was normalized to relative cell numbers. n = 9. *P < 0.05. (**E**) Immunoprecipitation analysis. 293 T-FlpIn-Flag-BEX2 cells were lysed and fractionated into mitochondria/nucleus and cytosol. (**F**) Oxygen consumption ratio (OCR) was determined under the treatment of siRNA of BEX2 and TUFM by Flux Analyzer. The data was normalized to relative cell numbers. (**G**) Tumorigenicity assay. Control or TUFM knock down HuCCT1 cells were injected into NOG mice subcutaneously (10^2^ cells per site. Six sites total.). Tumor volumes were measured weekly. (**H**) The sensitivity against gemcitabine was determined by MTT assay. The results were normalized to no gemcitabine treatment. n = 3. *P < 0.05 between siControl and siTUFM#1 or siTUFM#2. All the bars indicated means standard deviation.

To screen for the binding partners of BEX2 broadly, we performed another immunoprecipitation assay using a GST pull-down screen and detected other proteins (Fig. 6A), including HSPD1, TUFM, IVD and PECR, all of which are known mitochondrial proteins. We detected the binding partners HSPD1, TUFM, and IVD from both types of cell lysates (mouse kidney cells and HuCCT1 cells), suggesting that these proteins possess high specific affinity to BEX2 proteins. To clarify whether BEX2 is involved in mitochondrial function, we measured mitochondrial oxygen consumption ratio (OCR) using a flux analyzer in BEX2-knockdown cells (Supp Fig. 1C and 1D). The OCR was significantly increased by BEX2-knockdown in HuCCT1 cells (Fig. 6B). The extracellular acidification rate (ECAR) was significantly altered in siBEX2#2 but not in siBEX2#1 cells (Supp. Fig. 4A) We measured the intracellular ATP level to confirm the alteration of mitochondrial activity; the ATP level was significantly increased by BEX2-knockdown, which is consistent with our flux analyzer results (Fig. 6C). We also prepared knockdown cells for each of the four mitochondrial proteins (Supp Fig. 1E–H) and measured OCR. The OCR increased in all knockdown cell lines (Fig. 6D), whereas the ECAR was altered significantly in two mitochondrial protein knock down (PECR and TUFM), but not in other protein knock down (HSPD1 and IVD) (Supp. Fig. 4B). We confirmed the protein–protein interaction between BEX2 and these mitochondrial proteins using 293 T cells, and TUFM clearly bound BEX2 proteins (Fig. 6E). When both BEX2 and TUFM were knocked down, OCR, but not ECAR, increased similar to that in the single BEX2- or TUFM-knockdown lines (Fig. 6F, Supp. Fig. 4C), suggesting that BEX2 and TUFM cooperatively function in mitochondrial oxygen consumption. TUFM-knockdown cells had lower tumorigenicity and higher sensitivity against gemcitabine compared to control cells (Fig. 6G,H, Table 2). These data indicate that BEX2 suppresses mitochondrial function through its interaction with the TUFM mitochondrial protein.

**Table 2:** Tumorigenicity assay. Control or TUFM knock down HuCCT1 cells were injected into NOG mice subcutaneously (10^2^ cells per site. Six sites total).

Weeks	2	4	6	8	10	12
siControl	0/6	0/6	0/6	1/6	3/6	4/6
siTUFM#1	0/6	0/6	0/6	1/6	1/6	2/6
siTUFM#2	0/6	0/6	0/6	0/6	1/6	3/6

The number of palpable tumors were counted.

## Discussion

In the previous study, we found that cholangiocarcinoma dormant cancer stem cells had an enriched CD274^low^ cell fraction^6^. We analyzed differential gene expression in CD274-knockdown and control cells and identified BEX2, whose expression is mutually exclusive to CD274 expression in a variety of cancer cells, including cholangiocarcinoma cells. We then tested if BEX2 and CD274 regulate the expression of each other using siRNA against BEX2 or CD274, but did not find inverse regulation between the two genes (data not shown). However, a common regulator should be present in upstream of the BEX2–CD274 pathway that functions inversely in each pathway to induce G_0_ phase. Further study will be needed to elucidate this common regulator of the BEX2–CD274 pathway.

We demonstrated that BEX2 plays critical roles in the maintenance of dormant cancer stem cells in cholangiocarcinoma. In normal long-term hematopoietic stem cells, oxygen consumption and ATP content are lower than in whole bone marrow cells^18^, suggesting that mitochondrial activity is important for quiescent cell cycle. To investigate molecular mechanisms of the BEX2-mediated maintenance of dormant cancer stem cells, we screened for BEX2-binding proteins and identified TUFM, which is a translation elongation factor of tRNA^19^. After it is synthesized in the cytoplasm, TUFM is transported into the mitochondria, where it regulates expression of the mitochondrial genome by controlling the translation of mitochondrial DNA (mtDNA)-encoded proteins^20^. We show that BEX2-knockdown and TUFM-knockdown HuCCT1 cell lines have significantly increased mitochondrial OCR, indicating that BEX2 is a negative regulator of mitochondrial function and does so through interactions with TUFM. In contrast, the TUFM-knockdown in A549 cells decrease ATP levels^21^, which is not similar to our results presented here. We speculate that the mitochondrial function of the BEX2-TUFM complex could vary depending on cell type.

We also identified CUL2 and FEM1B as BEX2-binding proteins (Fig. 5). CUL2 is a member of the Cullin-RING ligases and interacts with a distinct family of substrate-receptor proteins to recruit ubiquitination targets^22^. CUL2 recruits and interacts with VHL-box proteins^23^, and FEM1B is a highly conserved VHL-box protein that has orthologues in metazoans^22^. There are three known substrates of FEM1B: TRA-1 in Caenorhabditis elegans^17^, Gli1 in Homo sapiens^24^, and Ankrd37 in Mus musculus^25^. In this study, we demonstrated that BEX2 is the fourth substrate of FEM1B-CUL2 E3 ubiquitin ligase complex. A previous study presented a fusion protein of nanobodies (i.e. natural single-domain antibodies containing only heavy chains) and SPOP, an adaptor protein of the Cullin-RING E3 ubiquitin ligase complex that is involved in rapid ubiquitination and subsequent proteasome-dependent degradation of specific nuclear proteins in mammalian cells^26^. In cholangiocarcinoma cells, we found that BEX2 was degraded within 3 h through its interaction with the FEM1B-CUL2 E3 ubiquitin ligase complex. Therefore, we hypothesize that agonistic compounds of the FEM1B-CUL2 E3 ubiquitin ligase complex could be suitable therapeutic tools that degrade BEX2 proteins in cholangiocarcinoma stem cells.

We found that BEX2 protein was completely degraded after only 3 h, even in the 293 T cells overexpressing BEX2, and treatment with proteasome inhibitors clearly inhibited BEX2 degradation, although endogenous BEX2 protein in a steady state is hard to detect by western blot. A previous report suggested that a low amount of enzyme in cells is a rate-limiting step of this degradation^27^. Thus, BEX2 is produced in cholangiocarcinoma cells and plays a critical role in dormant cancer stem cells.

In the present study, BEX2 was identified as a gene preferentially expressed in the CD274^low^ cell fraction that is enriched in G_0_ of dormant cancer stem cells in cholangiocarcinoma^6^. The analysis of the cell cycle phases after introduction or knockdown of BEX2 demonstrated that BEX2 is involved in the induction of the G_0_ phase in cholangiocarcinoma cells. In context, BEX2 is known to be expressed in a variety of tissues in fetal mice, and in Lgr5^+^ and Sox9^+^ stem/progenitor cells in the pyloric stomach and liver hepatic progenitor cells in BEX2-knockin mice^14^. Here, we found that the expression level of BEX2 is lower in mature cholangiocytes than in immature hepatoblasts. Although these data suggest that BEX2 is involved in the function of normal stem cells as well as cancer stem cells, our findings shed light on how controlling BEX2 can result in attenuation of CSC phenotypes and therapy resistance.

In conclusions, we demonstrated that BEX2 is essential for maintaining dormant cancer stem cells through the suppression of mitochondrial activity in cholangiocarcinoma. Our findings present BEX2-related mitochondrial pathway as a potential therapeutic target for intrahepatic cholangiocarcinoma.

## Materials and methods

### Ethics statements

This study was conducted according to the principles expressed in the Declaration of Helsinki and was approved by the Ethics Committees at Miyagi Cancer Center (Natori, Japan) and Tohoku University Graduate School of Medicine (Sendai, Japan). All procedures were approved by and executed in accordance with Miyagi Cancer Center and Tohoku University Graduate School of Medicine, and use committee regulations. The experimental protocols of animal experiments were approved by the Miyagi Cancer Center Animal Care and Use Committee (permit number: MCC-AE-2019-8).

### Cell lines

Intrahepatic cholangiocarcinoma cell lines HuCCT1 and RBE (RIKEN BioResource Center, Japan) were maintained in RPMI medium supplemented with 10% FBS and 1% penicillin–streptomycin. These cell lines were confirmed by short tandem repeat profiling. 293 T cells (RIKEN BioResource Center, Japan) were maintained in DMEM medium supplemented with 10% FBS and 1% penicillin–streptomycin. The U-251 (astrocytoma), MKN45 (gastric cancer), and BxPC3 (pancreatic cancer) cell lines were provided by the JCRB cell bank (Osaka, Japan). Huh7 cells were a gift from Dr. Chisari, (The Scripps Research Institute, CA). HepG2, Hep3B (hepatocellular carcinoma), Panc1 (pancreatic cancer), A549 (lung adenocarcinoma), LK2 (lung squamous cell carcinoma), and Lu99 (lung large cell carcinoma) lines were provided by the Institute of Aging and Cancer, Tohoku University. SH-SY5Y (neuroblastoma) and FaDu (pharyngeal cancer) cells were provided by the American Tissue Culture Collection (Manassas, VA). BICR6 cells were provided from the European Collection of Authenticated Cell Cultures (Salisbury, UK). 293 T-FlpIn-T-REx cells were purchased from Thermo Fisher Scientific (Waltham, MA USA). 293 T-FlpIn-T-REx-Flag-BEX2 cells stably express Flag-BEX2 in the presence of doxycycline and were established by introducing a pcDNA5/FRT vector containing a Flag-BEX2 insert into 293 T-FlpIn-T-Rex cells (Supplemental Fig. 2C). HuCCT1-BEX2 cells overexpressing human BEX2 were established from HuCCT1 cells using the PiggyBac system (System Bioscience, Palo Alto, CA) and selected for with puromycin (Sigma-Aldrich, St. Louis, MO). Cell lines were routinely tested for *Mycoplasma* contamination using MycoAlert detection kit (Lonza, Basel, Switzerland).

### Establishment of patient-derived xenograft (PDX) cell lines

Cholangiocarcinoma PDX cell line (CHOL4) was newly established by serial xenografts of tumor tissues derived from a human metastatic cholangiocarcinoma (adenocarcinoma) patient (62y, female) as described previously with minor modification^28^. In brief, fresh intrahepatic cholangiocarcinoma specimens were obtained from Tohoku University Hospital and transplanted into NOD/SCID/γc^null^ (NOG) mice (In-Vivo Science, Tokyo, Japan). Tumor formation was monitored weekly until they were over 10 mm in diameter, after which mice were sacrificed and the tumors were divided into three pieces for single-cell digestion, formalin fixation for histology, and serial passage in mice. The participant provided written informed consent.

### Short hairpin RNAs

To express the BEX2-specific short hairpin RNA (shRNA) in HuCCT1 and RBE cells, a retroviral vector was generated as described previously^29^. The target sequences were: #1: 5′-CAGTATAGATGGGACATAAT-3′ and #2: 5′-TTATGTCCCATCTATACTGT-3′. To express the CD274-specific short hairpin RNA (shRNA) in RBE cells, a retroviral vector was generated as described previously^6^.

### Plasmid construction

The PiggyBac-puro-BEX2 was constructed using a PCR-based cloning method. In brief, a cDNA encoding human BEX2 (RIKEN BioResource Center, Japan) was cloned and inserted in the PiggyBac-puro vector (System Bioscience, CA). PiggyBac- puro-BEX2 and a transposase vector was then transfected to HuCCT1 cells and positive transformants were selected for with puromycin resistance (HuCCT1-BEX2).

### Small interfering RNAs

Non-silencing control siRNA (12935-300), BEX2 siRNAs #1 (HSS131257) and #2 (HSS131258), FEM1B siRNAs #1 (HSS115379) and #2 (HSS115380), TUFM siRNAs #1 (HSS111058) and #2 (HSS111059), HSPD1 siRNA (HSS179400), IVD siRNA (HSS105619), PECR siRNA (HSS125122) were purchased from Invitrogen (Carlsbad, CA, USA). The siRNA transfections were performed using Lipofectamine RNAiMAX Reagent (Life Technologies, CA, USA) in antibiotic-free medium for 48 h. The siRNA knockdown efficiencies were confirmed by real-time PCR.

### Quantitative real-time PCR

Quantitative real-time PCR was performed as described previously^28^. In brief, total RNA was extracted from cell lines using an RNeasy Mini Kit (Qiagen, Valencia, CA) or Sepasol-RNA I Super G (Nakalai Tesque, Japan), and reverse transcribed using a PrimeScript II cDNA Synthesis Kit (Takara Bio, Japan). Real-time PCR was performed using the Brilliant III Ultra-Fast SYBR Green QPCR Master Mix (Agilent Technologies). β-actin was used as an endogenous reference gene. Primers used in this study are listed in Table S1.

### In vivo tumorigenesis

The tumor-formation assay was performed as described previously^6^. The cells were suspended in 50 μl DMEM supplemented with 10% FBS and an equal volume of Matrigel matrix (BD Biosciences) at 4 °C, and then injected into NOG mice with a 1-ml syringe. Tumor formation was monitored weekly.

### Gene expression profiling

Whole genome expression profiling of BEX2 or CD274-knockdown and control cells was performed as previously described^30^. Data processing were performed using R statistical software (version 3.6.1^31^) with a Rank Product package^32^. The dataset was uploaded to the Gene Expression Omnibus (GEO) database (GSE136741).

### Flow cytometry analysis

Cells were incubated with the appropriate antibody or mouse IgG control for 30 min at 4 °C, washed twice with PBS containing 3% FBS, and subsequently analyzed using a FACSCanto II (Becton Dickinson, CA). An Aldefluor kit (StemCell Technologies, Durham, NC) was used to isolate the population with a high aldehyde dehydrogenase (ALDH) enzymatic activity, according to the manufacture’s instruction.

### Cell cycle analysis

For cell cycle analysis, cells were trypsinized and then fixed with 70% ethanol at − 20 °C. The fixed cells were stained with anti-Ki67 (1:9) and 10 μg/ml propidium iodide (PI). The stained cells were then analyzed by flow cytometry.

### Western blotting

Western blotting was performed as described previously with minor modifications^30^. Cells were washed once with PBS without Ca and Mg, suspended in sodium dodecyl sulfate (SDS)-loading buffer (100 mM Tris–Cl pH 6.8, 4% sodium dodecyl sulfate, 0.2% bromophenol blue, 20% glycerol, 2% β-mercaptoethanol), sonicated for 5 min, boiled for 5 min, and then subjected to SDS-PAGE. The separated proteins were transferred onto a PVDF membrane (Millipore, Billerica, MA) and then blocked with 0.5% skim milk in TTBS (Tris-buffered saline with 0.1% Tween20) for 30 min at room temperature. The membrane was then incubated in 1:1000-diluted primary antibody and then in horseradish peroxidase (HRP)-conjugated anti-mouse or anti-rabbit antibodies (Cell Signaling Technology) as recommended by the manufacturer. Primary antibody binding was detected using a Clarity Western ECL Substrate (Bio-Rad, Richmond, CA), and images were captured by a CCD camera (Fuji Film, Tokyo, Japan).

### MTT assay

MTT assay was performed as described previously with minor modifications^30^. Cells were plated in 0.1 ml complete medium in a 96-well plate. At the indicated times, MTT (3-(4,5-di-methylthiazol-2-yl)-2,5-diphenyltetrazolium bromide) assay reagent (Roche, Basel, Switzerland) was added to each well according to the manufacturer’s protocol. The absorbance at 575 nm and 650 nm (background measurement) was determined using a plate reader (VersaMax ELISA Microplate Reader, Molecular Devices, Sunnyvale, CA, USA). Five replicate wells were assayed for each condition and the S.D. was determined.

### Luciferase assay

The BEX2 promoter region was amplified from the genome of HuCCT1 cells using a PCR method (Table S1). 293 T cells were transfected with pNL1.1-Nluc-Neo that included the BEX2 promoter sequence using FugeneHD (Promega, Madison, WI). Two days after transfection, cells were lysed with Nanoglo (Promega) and luminescence was measured using Synergy H1 (BioTek). pGL4.10[luc2] (Promega) was also transfected with pNL1.1-Nluc-Neo to normalize these values.

### Oxygen consumption ratio (OCR)

The OCR was measured using a Flux Analyzer XFe96 (Agilent, Santa Clara, CA). The cells were cultured in complete medium and the medium was replaced with DMEM containing Glutamax (Thermofisher) and 0.2% glucose during measurement. To normalize the OCR values with the exact cell number, relative cell number was confirmed with the plate after OCR measurement using sulforhodamine B-based in vitro toxicology assay kit as the manufacturer’s protocols (Sigma).

### Immunohistochemistry

Formalin-fixed paraffin embedded samples were sliced and deparaffinized. Antigen retrieval was performed using Immunosaver (NisshinEM, Tokyo, Japan). The slide was incubated with the first antibody anti-BEX2 antibody (C12, Santa Cruz, ×1000) and anti-Ki67 antibody (SP6, Abcam, ×800) at 4 ºC overnight. After washing with PBS, the slide was processed using Tyramide Signal Amplification Kits (Molecular Probe) for enhancing the signal of anti-BEX2 antibody. For anti-Ki67 antibody, anti-rabbit alexa594 antibody (Molecular Probe) was used as the secondary antibody. Nuclear staining was performed using 4′,6-diamidino-2-phenylindole (DAPI, Dojindo, Kumamoto, Japan). The slide was observed using a confocal microscopy (A1, Nikon, Tokyo, Japan).

### Nuclear extracts preparation

Nuclear extracts preparation was performed as previously described with minor modifications^33^. Crude nuclear fractions were prepared from HEK293 cells and kidney samples by homogenization in SHE buffer (10 mM HEPES pH 7.4, 0.21 M mannitol, 0.07 M sucrose, 0.1 M EDTA, 0.1 M EGTA, 0.15 mM spermine, 0.75 mM spermidine). The supernatant was collected by centrifugation (900×*g*, 10 min) was recentrifuged (2000×*g*, 10 min). The pellets containing the crude nuclear material were suspended in nuclear extraction buffer (50 mM HEPES pH 7.4, 0.3 M NaCl, 0.2% NP40, protease inhibitor cocktail complete (Roche) and sonicated for 15 s. The suspension was centrifuged again (12,000×*g*, 10 min) and the supernatant was used as crude nuclear extracts.

### Immunoprecipitation

Immunoprecipitation was performed as described previously^33^. HEK293 cells stably expressing Flag-BEX2 (1 ml) and the control cells expressing the induced pcDNA5/FRT/TO vector only (1 ml) were suspended in extraction buffer (50 mM HEPES pH 7.4, 0.3 M NaCl, 0.2% NP40) and sonicated for 5 min. The cell lysates were clarified by centrifugation at 10,000*g* for 30 min at 4 °C. The supernatants were filtrated through a Minisart syringe filter (Sartorius) and incubated with anti-flag antibody M2 beads (40 ml, sigma-Aldrich) for 4 h in the presence of Benzonase nuclease (10 mg/ml, Millipore) at 4 °C. After washing three times with washing buffer (0.15 M NaCl, 0.1% NP-40, 50 mM HEPES pH 7.4) and once with PBS, the binding proteins were eluted in 40 ml 0.1 M glycine buffer, pH 3.0. The eluates were neutralized with 4 ml 1 M Tris–HCl buffer pH 9.5 and suspended in SDS-PAGE (poly-acrylamide gel electrophoresis) sample buffer. The samples were boiled for 5 min and resolved by SDS-PAGE. The gel was stained using a mass silver stain kit (Wako, Osaka, Japan).

### GST pull-down

GST-BEX2 and deletion mutant constructs were inserted into the pGEX4T3 vector by PCR amplification and expressed in *E. coli*. Cell lysates from GST- BEX2 and deletion mutant constructs were incubated with glutathione magnetic beads (30 ml) for 1 h at 4 °C and then washed three times by washing buffer A (50 mM Tris–HCl pH 7.5, 0.3 M NaCl, 0.1% NP-40). The crude nuclear extracts were incubated with GST (control) and GST-BEX2-bound magnet beads for 12 h in the presence of Benzonase nuclease (Millipore, MA, USA) at 4 °C. After washing three times with washing buffer (0.15 M NaCl, 0.1% NP-40, 50 mM HEPES pH 7.4), GST (control) and GST-BEX2-bound proteins were eluted twice with 300 ml elution buffer (1.2 M NaCl, 50 mM HEPES pH 7.4). The eluates were concentrated and desalted with Amicon ultra-4-10k centrifugal filter units (Millipore). The eluates were resolved by SDS-PAGE and stained using a mass silver stain kit (Wako) as previously described^33^.

### nanoLC/MS/MS

Gel slippage was reduced with 100 mM DTT and alkylated with 100 mM idoacetamide. After washing, the gels were incubated with trypsin overnight at 30 °C. Recovered peptides were desalted with Ziptip c18 (Millipore, Burlington, MA) and analyzed by nanoLC/MS/MS systems as previously described^34^.

### Statistical analyses

The significance of differences between the experimental groups was calculated using Student’s t test. A difference of *P* < 0.05 was considered statistically significant.

## Supplementary Information

Supplementary Information.

## Abbreviations

ALDH: Aldehyde dehydrogenase
BEX2: Brain expressed X-linked gene 2
CSC: Cancer stem cell
DAPI: 4′,6-Diamidino-2-phenylindole
EGFR: Epidermal growth factor receptor
HRP: Horseradish peroxidase
IgG: Immunoglobulin G
iPS: Induced pluripotent stem
JNK: C-Jun N-terminal kinase
LC/MS/MS: Liquid chromatography/tandem mass spectrometry
MTT: 3-(4,5-Di-methylthiazol-2-yl)-2,5-diphenyltetrazolium bromide
OCR: Oxygen consumption ratio
PAGE: Poly-acrylamide gel electrophoresis
PI: Propidium iodide
SDS: Sodium dodecyl sulfate
TBS: Tris-buffered saline
