# Supplementary material for: BEX2 suppresses mitochondrial activity and is required for dormant cancer stem cell maintenance in intrahepatic cholangiocarcinoma
Source: Sci Rep. 2020 Dec 9;10:21592. doi: 10.1038/s41598-020-78539-0 (PMC7725823; doi:10.1038/s41598-020-78539-0)
Supplement: Supplementary file 1 — Supplementary Information. [file 41598_2020_78539_MOESM1_ESM.pdf]

BEX2 suppresses mitochondrial activity and is required for dormant cancer stem cell maintenance in intrahepatic cholangiocarcinoma

Keiichi Tamai*[1], Mao Nakamura-Shima[2], Rie Shibuya-Takahashi[1], Shinnichiro Kanno[3], Akira Yasui[3], Mai Mochizuki[1], Wataru Iwai[4], Yuta Wakui[4], Makoto Abue[4], Kuniharu Yamamoto[5], Koh Miura[5], Masamichi Mizuma[6], Michiaki Unno[6], Sadafumi Kawamura[7], Ikuro Sato[8], Jun Yasuda[2], Kazunori Yamaguchi[2], Kazuo Sugamura[2], Kennichi Satoh[1, 9].

[1]Division of Cancer Stem Cell, Miyagi Cancer Center Research Institute, 47-1, Medeshima-Shiode, Natori, Miyagi, Japan.

[2]Division of Molecular and Cellular Oncology, Miyagi Cancer Center Research Institute, 47-1, Medeshima-Shiode, Natori, Miyagi, Japan.

[3]IDAC Fellow Research Group for DNA Repair and Dynamic Proteome Institute of Development, Aging and Cancer (IDAC), Tohoku University, Sendai 980-8575, Japan

[4]Department of Gastroenterology, [5]Surgery, [7]Urology, [8]Pathology, Miyagi Cancer Center 47-1, Medeshima-Shiode, Natori, Miyagi, Japan.

[6]Department of Surgery, Tohoku University Graduate School of Medicine, 1-1, Seiryo-cho, Aoba-ku, Sendai, Miyagi, Japan.

[9]Division of Gastroenterology, Tohoku Medical and Pharmaceutical University, 1-15-1, Fukumuro, Miyagino-ku, Sendai, Miyagi, Japan

Supplemental Figure 1

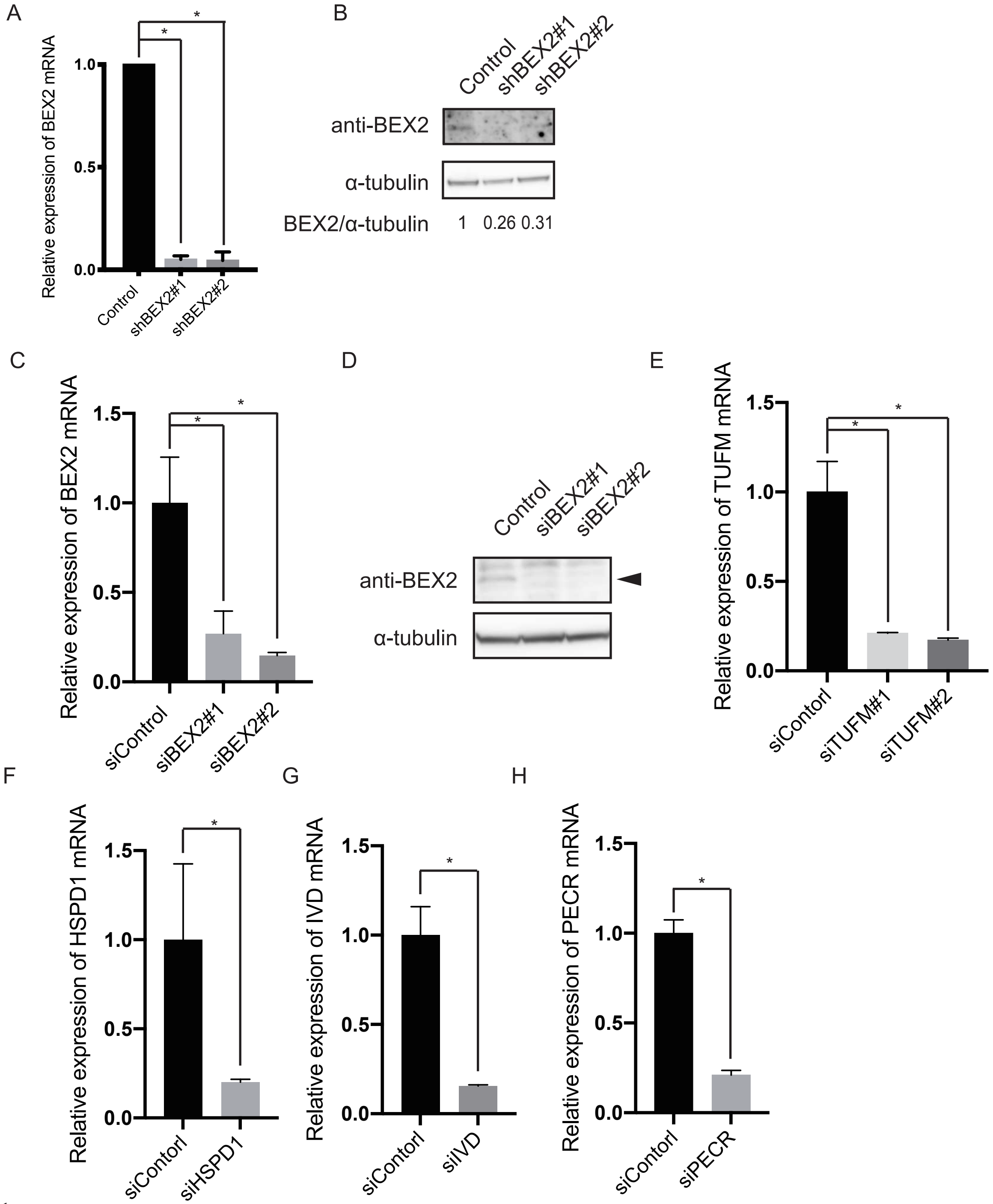

Supplemental Figure 1.

(A, B) Real-time PCR (A) and western blot analysis (B) of BEX2-knockdown and control HuCCT1 cells using the shRNA-expressing vector. In western blot analysis, cells were treated with 5 μM of MG132 for 24 h before harvest. The ratio of BEX2 and α-tubulin was measured by ImageJ software. (C, D) Real-time PCR (A) and western blot analysis (B) of BEX2-knockdown and control HuCCT1 cells by siRNA. In western blot analysis, cells were treated with 5 μM of MG132 for 24 h before harvest. (E, F, G, H) Real-time PCR of knock down HuCCT1 cells by indicated siRNAs. *P < 0.05.

Supplemental Figure 2

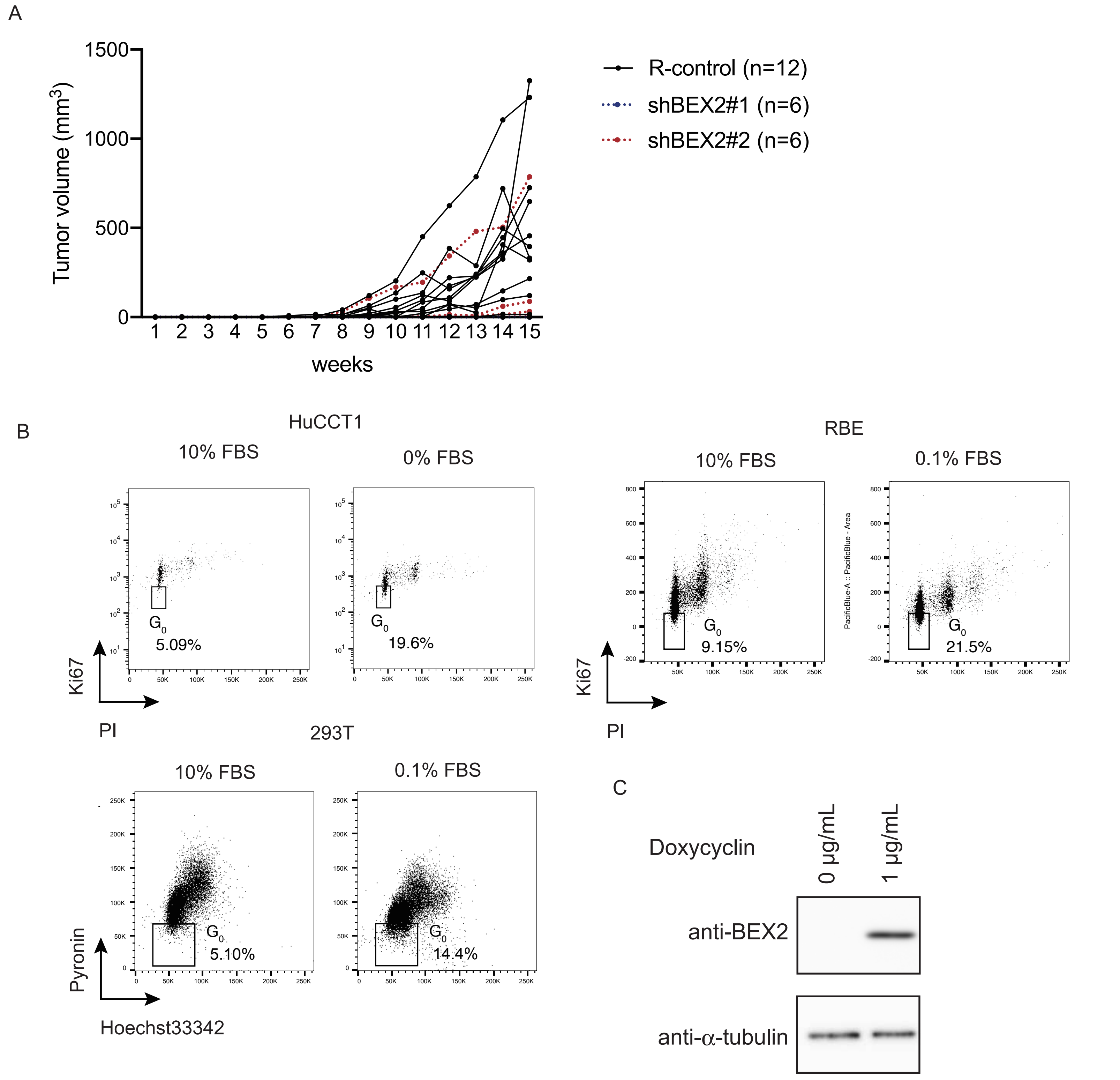

Supplemental Figure 2.

(A) The individual tumor volumes of Fig. 1B are plotted.

(B) Cells were starved under the medium containing 0 or 0.1% FBS for 96 h (HuCCT1), 48 h (RBE), and 24h (293T), the cell cycle was analyzed by flow cytometry. The cells were stained using Ki67 (proliferation marker) and propidium iodide (PI, DNA content), or pyronin (RNA content) and Hoechst33342 (DNA content). The open squares indicate the G_0 fracion.

(C) 293T-FlpIn-T-REx-Flag-BEX2 cells were treated with doxycyclin for 24h. Then the cells were harvested and the expression of BEX2 was examined by western blot.

Supplemental Figure 3

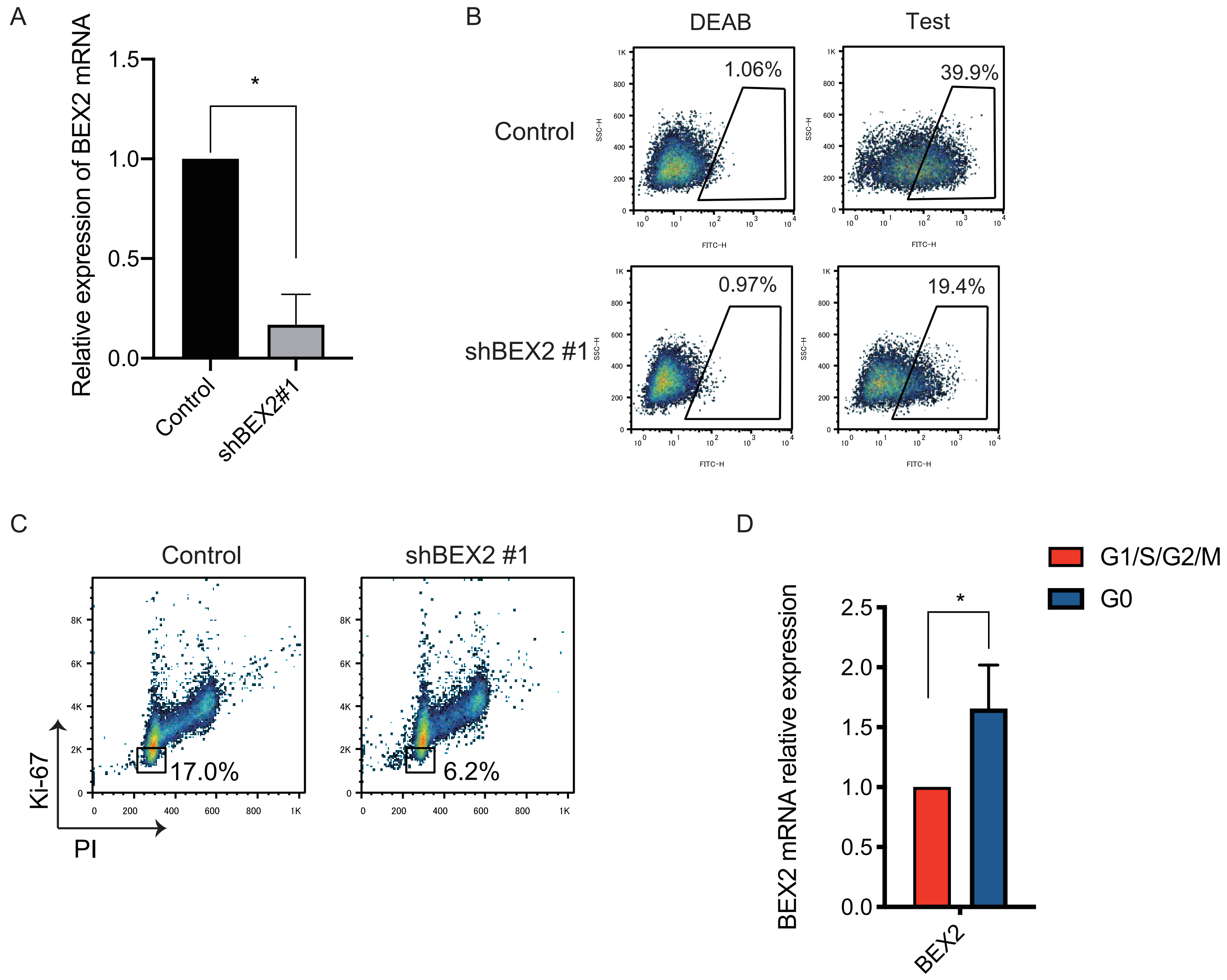

Supplemental Figure 3.
(A) Real-time PCR of BEX2-knock down and control RBE cells using the shRNA against BEX2. (B) ALDH activity was determined with an ALDEFLUOR assay in BEX2 knock down RBE cells. (C) Cell cycle assay of RBE cells. The cells were fixed with 70% ethanol and stained with PI and Ki67 for flow cytometry. G0 phase (open squre) was decreased in BEX2-knock down RBE cells. (D) Real-time PCR of sorted RBE cells.

Supplemental Figure 4

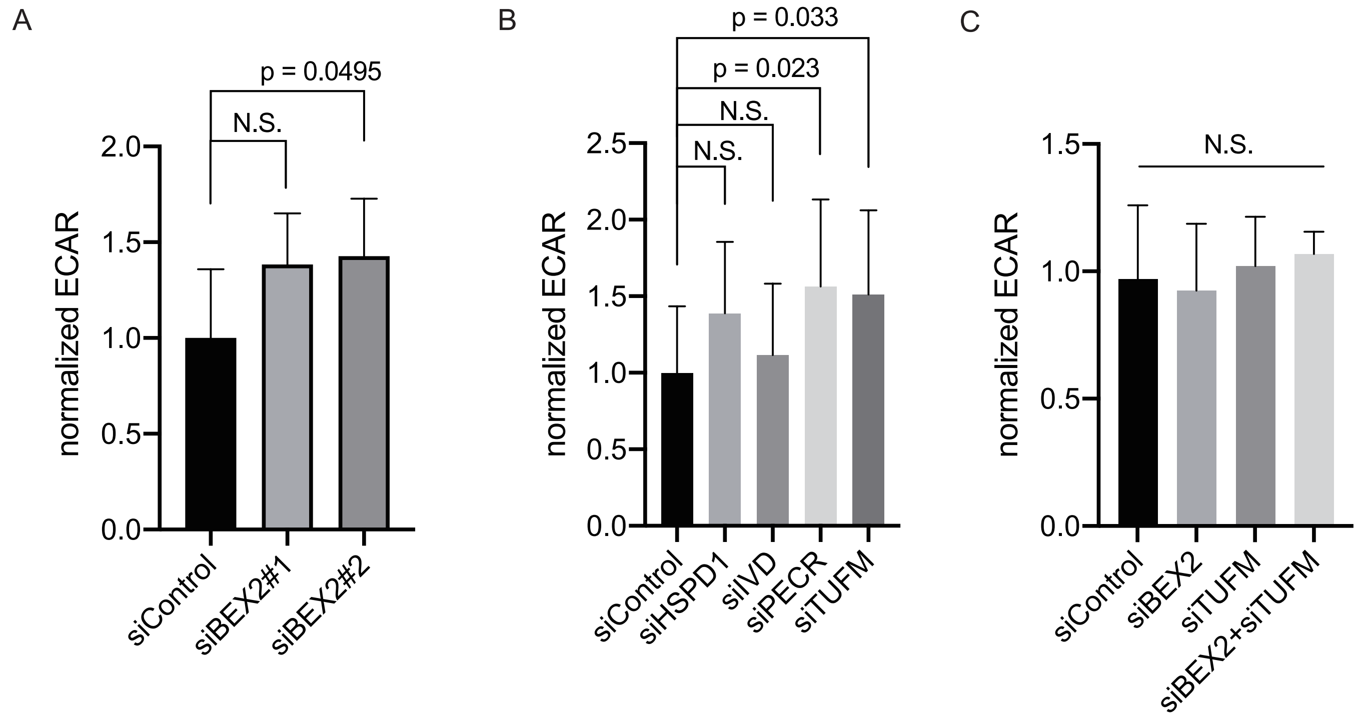

Supplemental Figure 4

(A to C) HuCCT1 cells were knocked down by siRNA, and ECAR was measured by flux analyzer. Graph bars indicate mean ± standard deviation. N.S., not significant.

Supplemental Figure 5

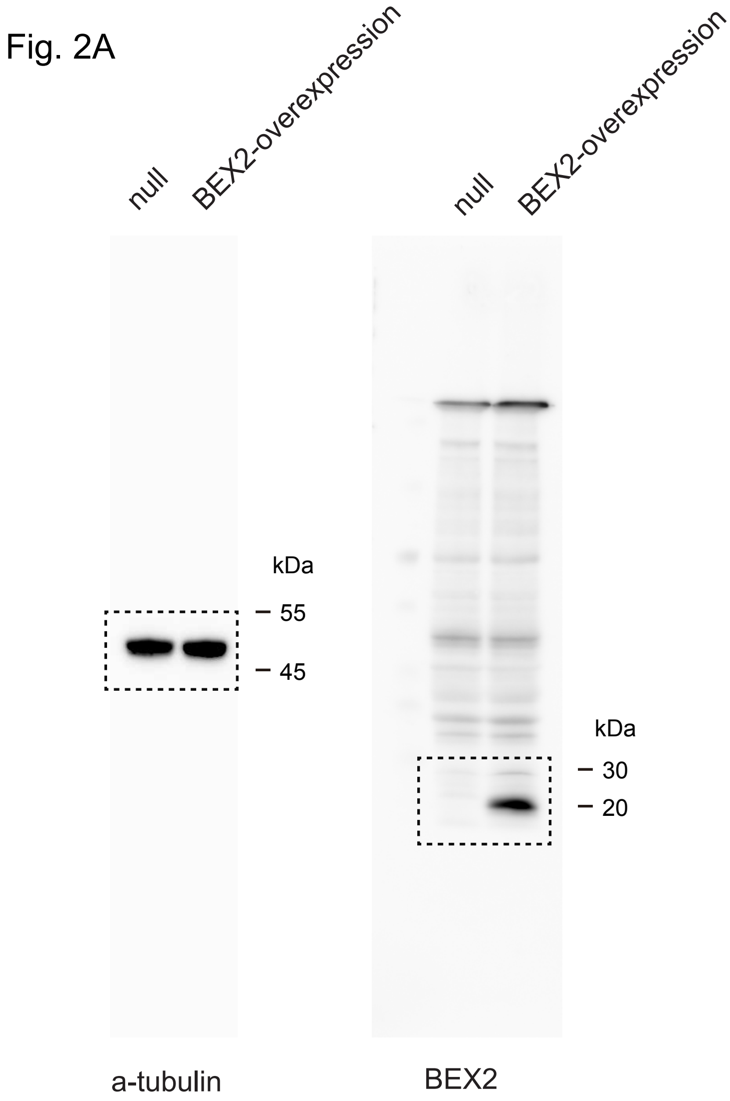

Supplemental Figure 5. Full size images of blots are shown.

Supplemental Figure 6

Fig. 5B

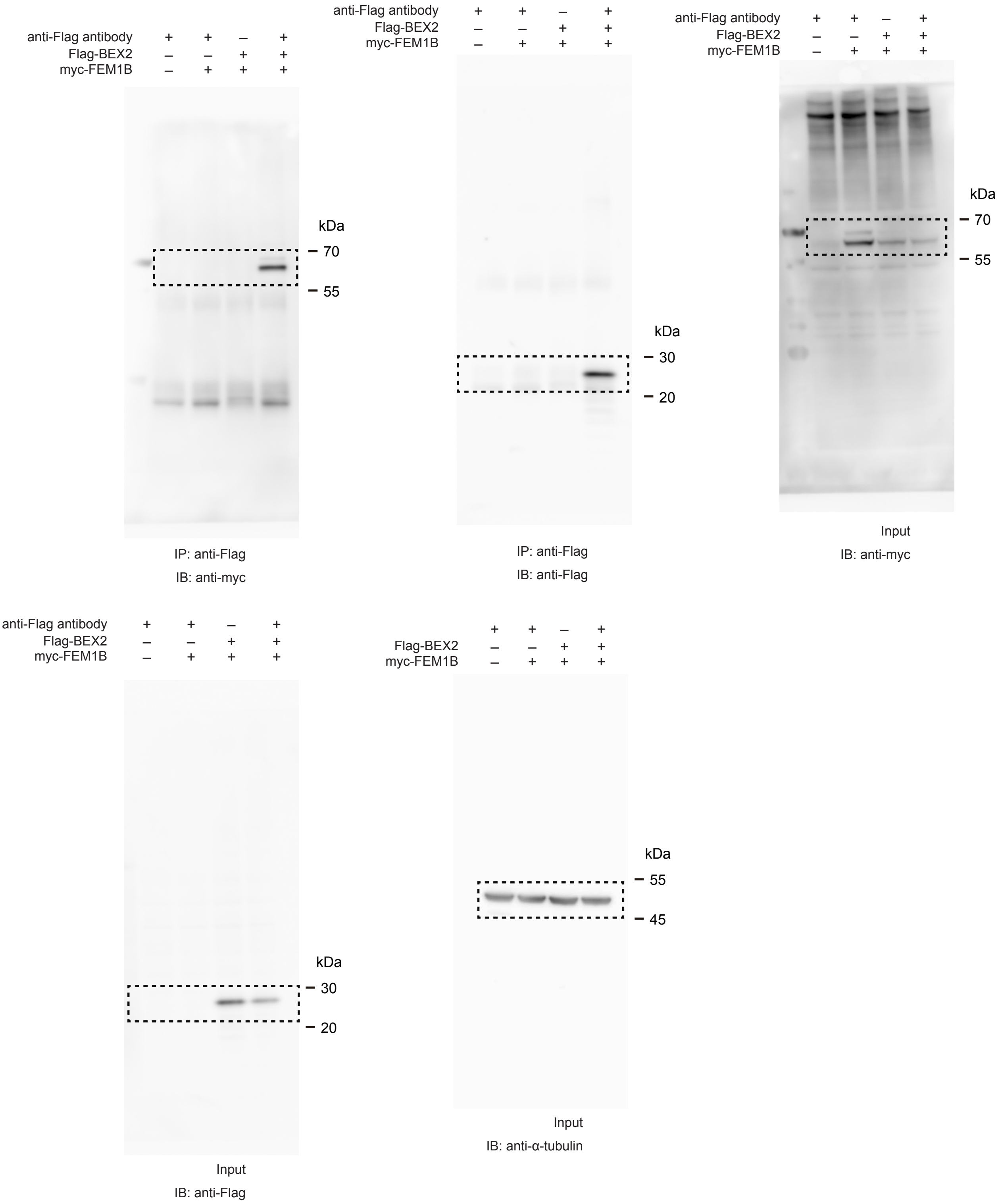

Supplemental Figure 6. Full size images of blots are shown.

Supplemental Figure 7

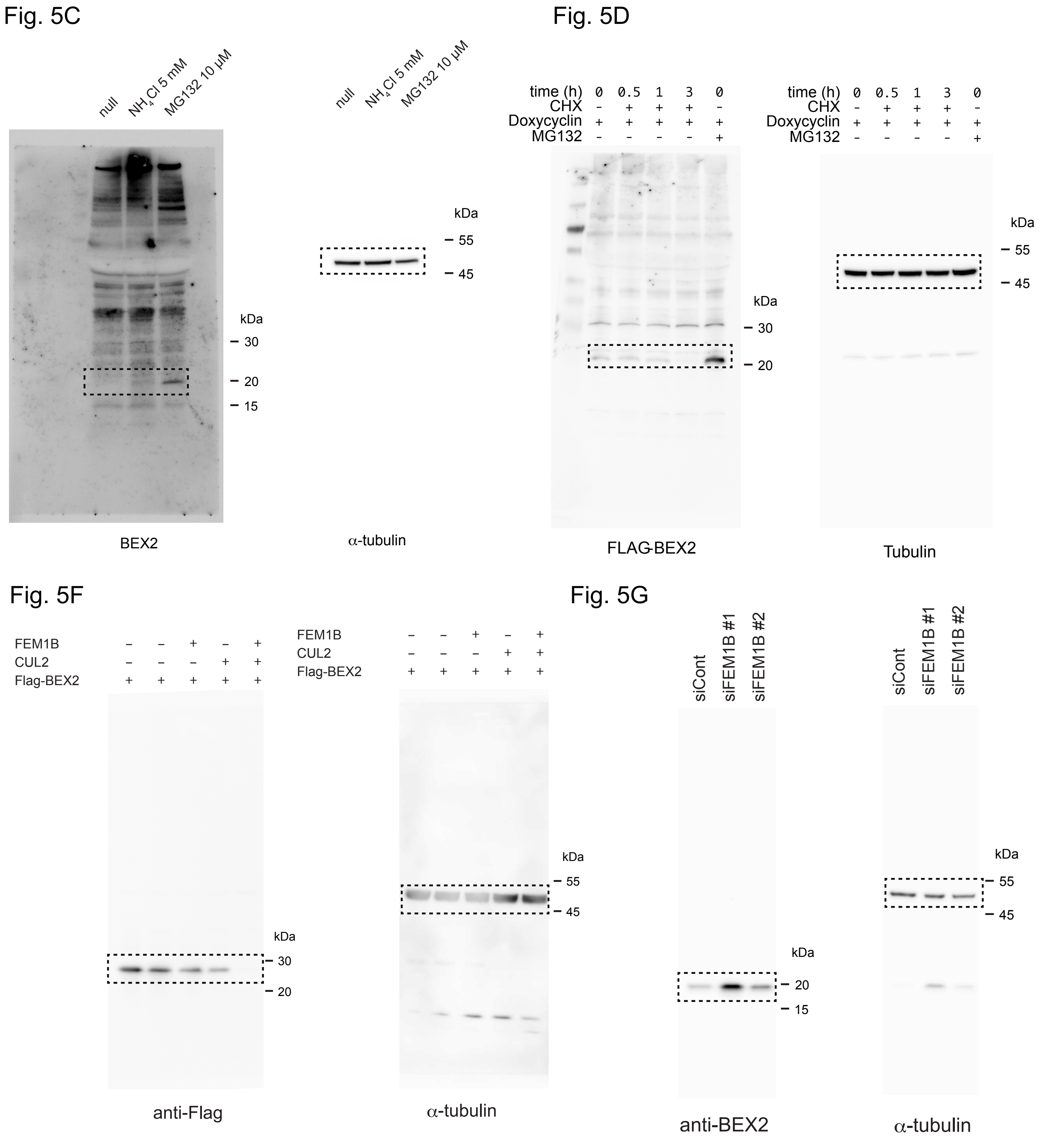

Supplemental Figure 7. Full size images of blots are shown.

Supplemental Figure 8

Fig. 6E

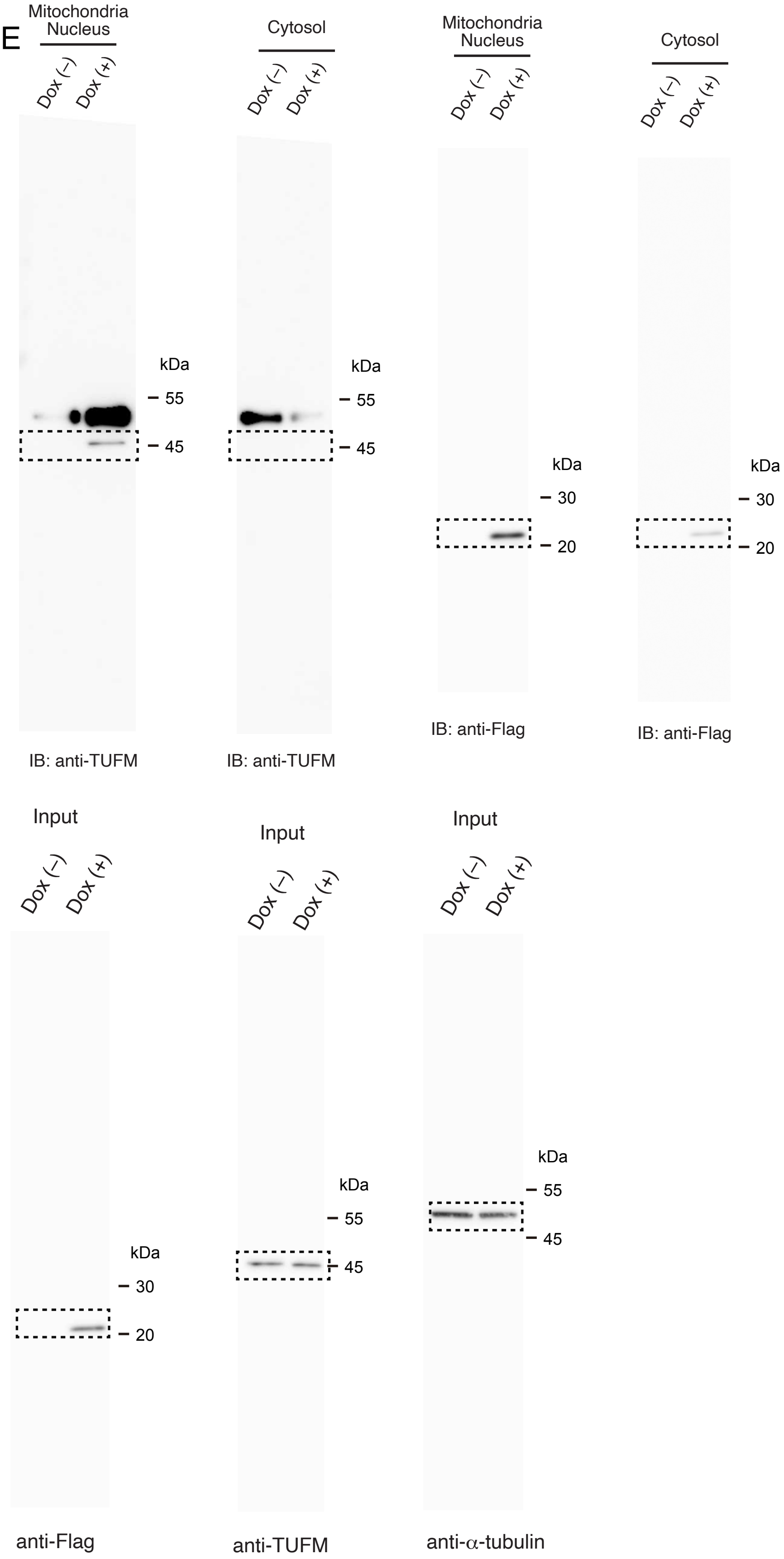

Supplemental Figure 8. Full size images of blots are shown.

Supplemental Figure 9

Supplemental Figure 1B

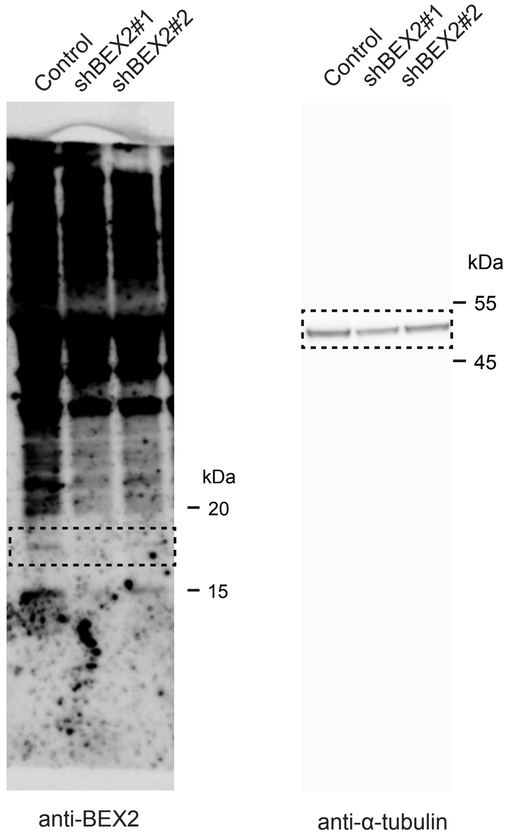

Supplemental Figure 1D

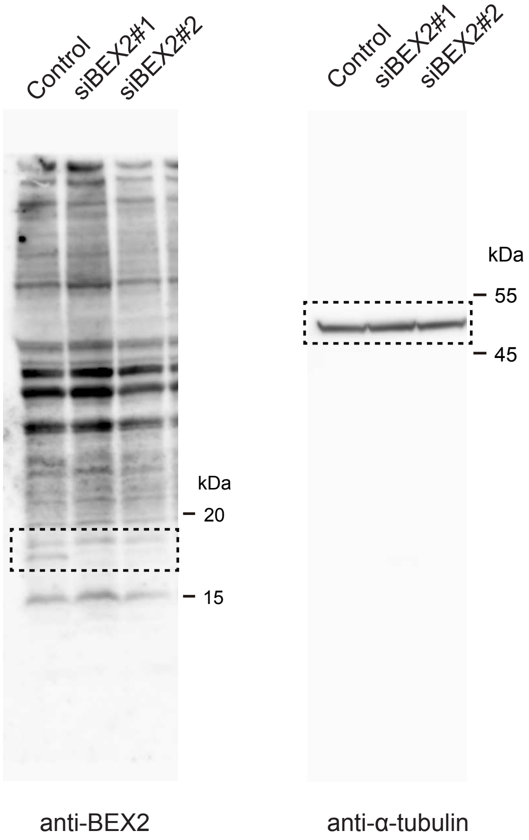

Supplemental Figure 2C

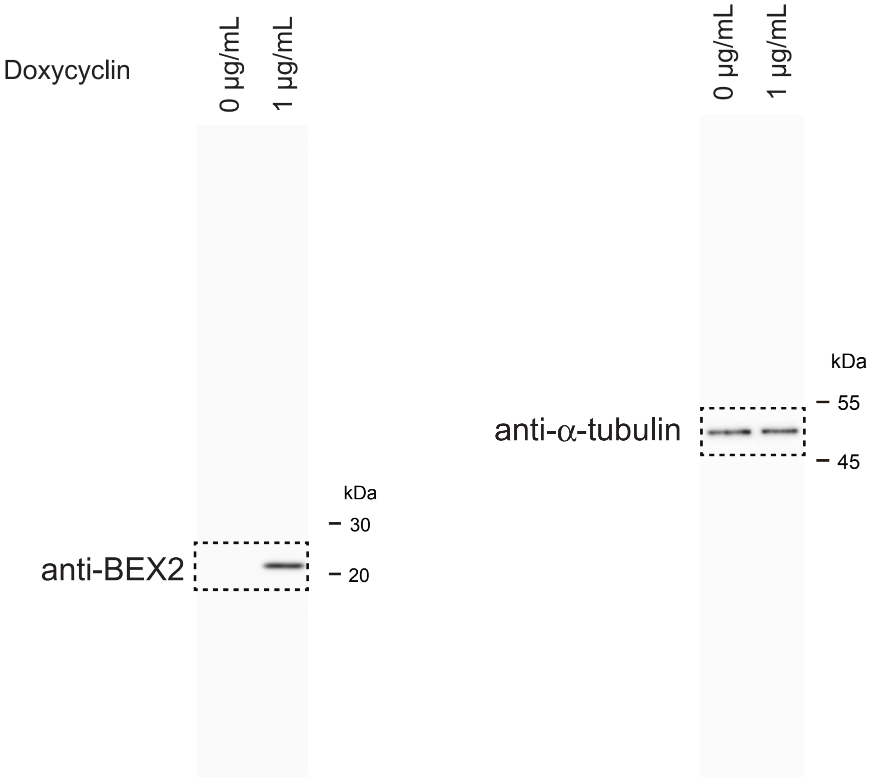

Supplemental Figure 9. Full size images of blots are shown.
